# Menstrual blood-derived mesenchymal stromal cell extracellular vesicles stimulate chondrocytes and cartilage extracellular matrix synthesis in vitro

**DOI:** 10.1038/s41598-026-40854-3

**Published:** 2026-02-25

**Authors:** Gabija Kugaudaite, Ieva Bakutyte, Edvardas Bagdonas, Raminta Vaiciuleviciute, Martynas Talaikis, Rokas Miksiunas, Ignas Lebedis, Jolita Pachaleva, Giedrius Kvederas, Edvinas Krugly, Eiva Bernotiene, Ilona Uzieliene

**Affiliations:** 1 Department of Regenerative Medicine, Innovative Medicine Centre, Santariskiu str. 5, Vilnius, 08406, Lithuania; 2 Life Sciences Center, Institute of Biochemistry, Vilnius University, Sauletekio av. 7, Vilnius, 10257 Lithuania; 3 Faculty of Medicine, Vilnius Santaros Clinics, Vilnius university, Santariškių str. 2, Vilnius, 08406, Lithuania; 4 Department of Environmental Technology, Kaunas University of Technology, Radvilenu Ave. 19, Kaunas, 50254, Lithuania

**Keywords:** Menstrual blood, mesenchymal stromal cells, extracellular vesicles, chondrocytes, cartilage explants, osteoarthritis, Biotechnology, Cell biology, Diseases, Medical research, Stem cells

## Abstract

Human mesenchymal stromal cells are frequently studied for the development of novel technologies for preventing osteoarthritis development or repairing cartilage tissue after traumas. Menstrual blood-derived mesenchymal stromal cells (MenSCs) are less studied, however, they possess a strong therapeutic potential due to their secretome, including extracellular vesicles (EVs). The aim of this study was to evaluate the potential of MenSC-EVs in stimulating chondrocyte functions, as well as cartilage tissue repair. Chondrocyte proliferation, morphology (holomonitor), sex hormone receptor expression (ELISA, RT-qPCR), and chondrogenic capacity (RT-qPCR, histology) were evaluated after 3 days of MenSC-EV treatment. Cartilage explants were isolated and treated with EVs for 3 days and cultured for 3 and 7 days under inflammatory (IL-1β) or regenerative (TGF-β3) conditions. Cartilage oligomeric matrix protein (COMP) secretion and glycosaminoglycan (GAG) release were assessed by ELISA and spectrophotometry, with extracellular matrix (ECM) deposition evaluated by histology/immunohistochemistry, infrared absorption spectroscopy and gene expression by RT-qPCR. Cytokine and growth factor secretion were quantified in explant and chondrocyte culture supernatants using multiplexed Luminex assays. MenSC-EVs did not affect chondrocyte migration/motility, speed/perimeter and proliferation after 7 days. However, EVs increased progesterone receptor expression, as well as ECM production together with collagen type II and TGF-β3 receptor gene expression in chondrocytes after 21 days with TGF-β3. Cartilage degradation was diminished after treatment with MenSC-EVs according to lower levels of COMP and GAGs released under conventional or inflammatory conditions. Moreover, MenSC-EVs did not significantly affect inflammatory cytokine secretion in cartilage explants stimulated or not with IL-1β and in chondrocyte monolayer, except for secretion of IL-6. In addition, cartilage ECM components (collagen II and aggrecan), as well as TGF-β3 receptor gene expression were significantly higher in MenSC-EV treated cartilage explant samples, as compared to non-treated. Infrared absorption spectroscopy of MenSC-EV treated cartilage explants corroborated ECM restoration, with samples exhibiting higher amide I/II intensities and a stronger carbohydrate-associated band near 1030 cm^− 1^, consistent with increased collagen II and proteoglycan content. This study demonstrates the potential of MenSC-EVs stimulating ECM synthesis in chondrocytes, which may turn out to be a promising cell-free therapy in the future.

## **Background**

Cartilage defects and traumas often lead the tissue to the development of osteoarthritis (OA), which is a chronic, progressive joint disorder characterised by the degradation of articular cartilage, subchondral bone remodeling, synovial inflammation, and loss of joint function. OA is one of the most prevalent musculoskeletal diseases worldwide, significantly affecting the quality of life and imposing a heavy socioeconomic burden^1^. Moreover, the prevalence and severity of OA are higher in women than in men, especially in weight-bearing joints such as the knees and hips. This sex-related difference becomes most pronounced during the postmenopausal period, when the incidence of OA highly increases^2,3^. Current clinical treatments are primarily aimed at symptom management, for instance reducing pain and inflammation, while no therapy exists to stop or reverse the degenerative process^4^. As such, regenerative medicine approaches have gained increasing attention for their potential to restore damaged cartilage and preserve joint function.

Among the strategies, human mesenchymal stromal cells (MSCs) have emerged as a promising tool due to their regenerative, immunomodulatory, and anti-inflammatory properties^5,6^. MSCs present their beneficial effects largely through paracrine mechanisms, mediated by a complex secretome that includes soluble factors, bioactive molecules, and extracellular vesicles (EVs)^7^. EVs, comprising exosomes and microvesicles, serve as natural carriers of proteins, lipids, and nucleic acids, enabling intercellular communication and modulation of tissue repair processes^8,9^. In recent years, cell-free therapies using MSC-derived EVs have been proposed as an alternative to direct MSC transplantation, offering advantages such as lower immunogenicity, reduced risk of tumorigenicity, and more straightforward storage and handling^8,10^. While bone marrow, adipose tissue, and umbilical cord–derived MSCs are extensively studied in the context of cartilage repair^11^, menstrual blood–derived MSCs (MenSCs) remain an underexplored, but highly promising source. MenSCs can be collected through a non-invasive and ethically acceptable procedure, enabling repeated sampling from healthy donors^12^. These cells possess high proliferative capacity, multipotency, and a rich secretome profile, making them attractive for regenerative applications^13,14^. Moreover, MenSC-derived EVs (MenSC-EVs) inherit many of the parent cell’s bioactive properties and have been shown to modulate inflammation, promote tissue regeneration, and influence the function of target cells^14,15^.

The potential application of MenSC-EVs in cartilage repair during OA is particularly intriguing. Articular chondrocytes, the only cell type within cartilage, are responsible for maintaining extracellular matrix (ECM) homeostasis^16^. In OA and after traumatic injury, chondrocytes undergo phenotypic changes, reduced anabolic activity, and increased catabolic enzyme production, leading to ECM breakdown^16,17^. Moreover, it is known that the development of OA in postmenopausal women is closely linked to the decline in sex steroid hormones, particularly estrogen and progesterone^18^. Chondrocytes, synoviocytes, and other joint tissues express estrogen receptors (ERα, ERβ) and progesterone receptors (PR), which are involved in regulating cartilage metabolism, ECM synthesis, and inflammatory responses^18–20^. Estrogen has been shown to enhance proteoglycan and collagen production, inhibit matrix-degrading enzymes, and exert anti-inflammatory effects, while progesterone contributes to chondrocyte viability and ECM homeostasis^20^.

If MenSC-EVs could enhance chondrocyte anabolic function, stimulate redifferentiation toward a cartilage-producing phenotype, protect ECM integrity under inflammatory stress, and/or influence the expression of sex hormone receptors, they could represent a novel therapeutic avenue for OA prevention and cartilage regeneration. However, to date, limited studies have evaluated the specific effects of MenSC-EVs on chondrocyte biology or cartilage tissue under conditions mimicking both homeostatic and inflammatory environments. **Therefore**,** the aim of the present study was to evaluate the potential of MenSC-EVs in stimulating chondrocyte functions and chondrogenic redifferentiation**,** as well as cartilage tissue repair.** During this study, we have investigated the uptake of MenSC-EVs by human chondrocytes, their influence on chondrocyte proliferation, metabolic activity, morphology, sex hormone receptor expression, and chondrogenic redifferentiation, as well as their effect on cartilage explants cultured under regenerative (transforming growth factor β3 (TGF-β3)) and inflammatory (interleukin 1β (IL-1β)) conditions.

The findings of this study offer new insights into the regenerative and protective capacities of MenSC-EVs and highlight their potential as a cell-free therapeutic strategy for cartilage tissue repair and OA prevention, while also emphasizing their unique relevance as a previously overlooked, non-invasive stem cell source with high translational potential.

## Methods

### Cell isolation and culture

Menstrual blood samples were collected from 3 healthy donors (age 27/32/34) during the 2nd day of menstruation. About 5–10 mL of menstrual blood was collected by donors using sterile silicone cups (iCare, Wuhan, China). MenSCs were separated using Ficoll-Paque PLUS (Stem Cell Technologies, Cologne, Germany) density gradient centrifugation (30 min, 400 *g*) and washed out two times in phosphate-buffered saline (PBS) (Sigma Aldrich, Taufkirchen, Germany), then centrifuged (10 min, 600 *g*). MenSC phenotypic characterization according to established MSC criteria (plastic adherence, trilineage differentiation, surface marker expression) was previously demonstrated^21^.

Post-surgical cartilage samples from 10 female donors (age range 65–85, OA grade 3–4) were collected at Vilnius University Santaros hospital (Vilnius, Lithuania). Chondrocytes were isolated from the tissue using enzymatic digestion by pronase (Roche diagnostics, Basel, Switzerland), followed by type II collagenase (Biochrom AG, Berlin, Germany), as previously discussed in^22^.

Collected cells were seeded into tissue culture flasks (Gibco, Thermo Fisher Scientific, Waltham, MA, USA) with low glucose (1 g/L) Dulbecco’s modified Eagle medium (DMEM) (Merck Millipore, Darmstadt, Germany) supplemented with 10% fetal bovine serum (FBS) (Merck Millipore, Darmstadt, Germany), 1% penicillin/streptomycin (PS) (Gibco, Thermo Fisher Scientific, Waltham, MA, USA) (later referred as “complete medium”), and cultured in a cell culture incubator with 37 °C and 5% CO_2_. Medium was changed twice a week, and after cells reached confluence of ~ 80%, they were detached using a 0.25% solution of trypsin-EDTA (Gibco, Thermo Fisher Scientific, Waltham, MA, USA), counted and sub-cultured. Early passages (up to *p* = 3) of MenSCs and chondrocytes were used in experiments. Early passages (≤ P3) were used to minimize phenotypic drift and preserve MSC characteristics of MenSCs. All procedures using human donor tissues in this study were performed in compliance with relevant guidelines and regulations (Bioethical Permissions No. 158200-14-741-257 and No. 2021/6-1363-837), and approved by the Vilnius Regional Biomedical Research Ethics Committee. Informed consent forms were signed and obtained from all human tissue donors prior to sample collection.

### EV isolation from MenSCs

The isolation of MenSCs-EVs was performed using size exclusion chromatography (SEC) system Izon. The supernatant proteins are first concentrated using (100 kDa) Amicon ultra centrifugal filters (Merck Millipore, Darmstadt, Germany) and centrifugation (55 min, 3000 *g*). The resulting concentrate is diluted with filtered, sterile Dulbecco’s phosphate-buffered saline (DPBS) up to 500 µL and loaded onto qEVoriginal 70 nm GEN2 chromatography columns (Izon Science, Christchurch, New Zealand) according to the manufacturer’s protocol. The isolated EVs are then analysed by flow cytometry.

### EV quantification and characterisation

EV quantification was performed using CytoFLEX LX flow cytometer (Beckman Coulter, Brea, CA, USA). The cytometer was deep cleaned before the event analysis and Apogee beads (Apogee Flow, Hemel Hempstead, UK) were used to detect the specific region of EV population (from 80 nm to 1300 nm). Events were gated on the VSSC-width log x VSSC-H log cytogram to remove EV aggregates (singlet gate). A rectangular gate for stable time of the collected events was set from 90 s to 150 s and a rectangular gate for the events of interest was set on the VSSC-H log x RSSC-H log cytogram containing the EV population. To avoid swarm effects each was serially diluted from 1:2 to 1:10 to achieve an event count of 5000 events/s and measured with a flow rate of 10 µl/min. The EV count was determined as the events/µl within the microparticle region.

For EV characterisation, EV markers (CD63, CD81 and CD9) were detected by EV-Bead Conjugated Flow Cytometry on the CytoFLEX LX flow cytometer. 12 × 10^6^ EVs/test was mixed with 0.1 µl/test aldehyde/sulfate latex beads (4 μm; Thermo Fisher Scientific, Waltham, MA, USA) in 200 µl PBS rotating overnight at 4 °C. For negative controls, beads without EVs were stained under the same conditions with the same antibodies. The beads were blocked in 1% bovine serum albumin (BSA) for 1 h at room temperature (RT). The samples were then washed with PBS three times and the beads were resuspended in 1% BSA. The beads were stained with anti-CD9-phycoerythrin (PE), CD63-PE, anti-CD81-PE (BD Biosciences, Franklin Lakes, NJ, USA) antibodies for 30 min on ice and then washed with PBS three times. Flow cytometry analysis was performed on the CytoFLEX LX flow cytometer, with the gating of EV-decorated 4 μm diameter beads being based on FSC/SSC parameters.

### EV uptake by chondrocytes in monolayer

EV uptake was performed by seeding chondrocytes at a density 50,000 cells per well in 6 well plates in 2 mL complete medium. The next day, EVs were stained with 20 mM of carboxyfluorescein diacetate, succinimidyl ester (CFDA) fluorescent membrane dye (Invitrogen, Thermo Fisher Scientific, Waltham, MA, USA) for 30 min in a cell culture incubator (37 °C and 5% CO_2_), after performing SEC for depleting the unbound dye. In parallel, the same concentration of CFDA dye in DPBS was prepared under the same conditions as an unbound dye control. The prepared EV-CFDA and DPBS-CFDA were analysed by flow cytometry (CytoFLEX LX), evaluating the concentration of EVs and staining % by B525 (CFDA emission) channel, after subtracting DPBS-CFDA result, and added to the seeded chondrocytes (100 EV/cell). EV doses were normalized to the number of recipient cells present at the time of treatment, ensuring a consistent vesicle-to-cell ratio across all experiments. After 3 h, the chondrocytes were detached and measured by flow cytometry (CytoFLEX LX) in order to evaluate the EV uptake by cells.

### Chondrocyte proliferation assay

Chondrocytes were seeded at a density of 5,000 cells per well into 12-well plates in 1 mL of complete medium. Cells were incubated for 24 h in a cell culture incubator (37 °C and 5% CO_2_) to allow attachment. After, 100 EVs/cell were added to the 6 wells of cultured chondrocytes, supplementing other 6 wells with the same amount of DPBS (EV dilution buffer) and keeping as control. Cell proliferation was assessed on days 3 and 7 using the Cell Counting Kit-8 (Dojindo Europe GmbH, Munchen, Germany). After 3 days of incubation with EVs, the medium was collected for further multiplex assay, while replaced fresh medium did not contain additional EV portions.

At each time point, the culture medium was replaced with a CCK-8 working solution, prepared by mixing 40 µL of CCK-8 reagent with 1 mL of complete medium. Cells were incubated with the solution for 3 h at 37 °C in a 5% CO₂ incubator. After incubation, 100 µL of the supernatant from each well was transferred into a 96-well plate and the absorbance was measured at 450 nm using a SpectraMax i3 microplate reader (Molecular Devices, San Jose, CA, USA). Each well was measured in triplicates (three technical replicates per donor), and background absorbance was corrected using blank controls (medium with CCK-8, without cells).

### Chondrocyte migration, motility speed and perimeter assay

Chondrocyte migration, motility speed and perimeter analysis is performed using the Holomonitor instrument (Phiab, Lund, Sweden), applying Single Cell tracking protocol and analysed using the Holosuit program. Before the assay, chondrocytes are seeded into 6 well plates at a density 10,000 cells/well and cultured overnight in a cell culture incubator to allow attachment. The next day, 3 wells are supplemented with 100 EVs/cell, while other 3 wells are supplemented with the same amount of DPBS (EV dilution buffer) and kept as control. After, the plate with chondrocytes is covered with hololids and transferred to the holomonitor for cell observation and focusing. At least 6–8 different focus points/ positions are selected from each well for further cell monitoring and image capture. Images from every position are captured for 24 h.

Chondrocyte analysis was performed after all images have been captured, selecting the cells from every position image manually and exporting the numerical values of migration, motility speed and perimeter after 24 h, which are automatically measured by the software.

### Immunoabsorbent assays (ELISA) for estrogen receptor alpha 1 and progesterone receptor expression in chondrocyte monolayer

To assess intracellular expression of estrogen receptor alpha (ESR1) and progesterone receptor (PGR), chondrocytes were seeded at a density of 50,000 cells per well into 6-well culture plates with 2 mL of complete medium. After 24 h of attachment, 3 wells are supplemented with 100 EVs/cell, while other 3 wells are supplemented with the same amount of DPBS (EV dilution buffer) and kept as control. Chondrocytes were cultured for an additional 3 days in a cell culture incubator (37 °C, 5% CO₂). After 3 days, 2 control and 2 EV treated well cells were washed twice with ice-cold PBS, followed by three freeze–thaw cycles (− 80 °C to room temperature) to ensure complete cell lysis, while 1 control and 1 EV-treated well were lysed using RLT lysis buffer supplemented with 10% β-mercaptoethanol for further RT-qPCR evaluation of ESR1 and PRG receptor gene expression. Lysates were collected and stored at − 20 °C until analysis. Before ELISA, samples were thawed on ice and total protein concentration was determined using the BCA Protein Assay Kit (Cell Signaling Technology, Danvers, MA, USA), as discussed in method Sect.  2.13. Protein concentrations were normalized across samples. Quantification of ESR1 and PGR levels in cell lysates was performed using commercial ELISA kits from ELK Biotechnology (Wuhan, China). All steps were carried out according to the manufacturer’s protocols. Absorbance was measured at 450 nm using a SpectraMax i3 microplate reader. All samples were measured in triplicates and results were expressed relative to total protein concentration (e.g., pg/ng per µg protein).

### Chondrocyte monolayer protein normalization assay

Total protein concentration in chondrocyte lysates was quantified using the BCA Protein Assay Kit, according to the manufacturer’s protocol. Briefly, samples were diluted as needed, mixed with the BCA working reagent in a 96-well plate, and incubated at 37 °C for 30 min. Absorbance was measured at 562 nm using a SpectraMax i3 microplate reader. A standard curve was generated using BSA standards (0–2000 µg/mL), and sample protein concentrations were interpolated from the curve. Total protein values were used to normalize downstream ELISA analyses.

### Chondrogenic induction study

A total of 200,000 chondrocytes per well were transferred into round-bottom, low-attachment 96-well plates. Cells were centrifuged at 500 *g* for 5 min to initiate pellet formation. The culture medium was replaced with chondrogenic differentiation medium, consisting of high-glucose DMEM (4.5 g/L) with 1% PS, 1% insulin-transferrin-selenium (ITS) (Gibco, Thermo Fisher Scientific, Waltham, MA, USA), 0.35 mM L-proline (Carl Roth, Karlsruhe, Germany), 10⁻⁷ M dexamethasone, and 0.17 mM ascorbic acid phosphate (Sigma Aldrich, Taufkirchen, Germany). The following experimental groups of chondrogenic pellets were established:

- Chondrogenic medium only (no growth factors).
- EVs only: 100 EVs per cell, added once at day 1.
- Transforming growth factor β3 (TGF-β3) only: 10 ng/mL (Thermo Fisher Scientific, Waltham, MA, USA).
- TGF-β3 + EVs: 10 ng/mL TGF-β3 and 100 EVs per cell (EVs added once at day 1).

In all groups where EVs were applied, the dose of 100 EVs per cell was added once at the beginning of differentiation (day 1). The pellets were cultured for 21 days, with chondrogenic medium refreshed three times per week. Throughout this period, cell aggregates formed visible pellets. At the end of the differentiation protocol, pellets were harvested for downstream analyses: RT-qPCR and histological staining.

### Cartilage explant isolation and culture

Post-surgical cartilage samples from previously described donors (10 female donors (age range 65–85, OA grade 3–4)) at Vilnius University Santaros Hospital (Vilnius, Lithuania) were used for explant isolation. The cartilage tissue was washed with PBS + 2% PS, excised from the bone under sterile conditions using a scalpel and incubated in 1 g/L glucose DMEM supplemented with 2% PS placed in a cell culture incubator (37 °C and 5% CO_2_) overnight. The next day, cartilage samples were processed and cut into 3 mm diameter explants by biopsy punch needle and collected in a sterile Petri dish with 1 g/L glucose DMEM with 1% PS, incubating them (37 °C and 5% CO_2_). After, cartilage explants were transferred to x3 6 well plates (120 mg of explants/well) and supplemented with chondrogenic differentiation medium (composition discussed above). One plates 3 wells are supplemented with 100 EVs/chondrocyte, while other 3 wells are supplemented with the same amount of DPBS (EV dilution buffer) and kept as control. Second plates 3 wells are supplemented with TGF-β3 (10 ng/mL), while other 3 wells are supplemented with TGF-β3 (10 ng/mL) + EVs (100 EV/cell). Third-plate 3 wells are supplemented with interleukin-1β (IL-1β) (10 ng/mL) (Thermo Fisher Scientific, Waltham, MA, USA), while other 3 wells are supplemented with IL-1β (10 ng/mL) + EVs (100 EV/cell).

After 3 days, the medium is collected for further analysis and replaced with fresh chondrogenic medium without EVs/DPBS, but with adding TGF-β3 or IL-1β. After 7 days, the medium is collected for further analysis and cartilage explants are analysed using histological/immunohistochemical (IHC) staining. Cartilage explants treated or not with EVs were additionally evaluated using RT-qPCR.

### RNA isolation

#### Chondrocyte pellets

After the 21-day chondrogenic differentiation period, chondrocyte pellets were washed twice with PBS and homogenized using a syringe in RLT lysis buffer supplemented with 10% β-mercaptoethanol, according to the protocol provided with the RNeasy Mini Kit (Qiagen, Venlo, The Netherlands). Total RNA was extracted using RNeasy Mini Spin Columns (Qiagen, Venlo, The Netherlands) following the manufacturer’s instructions.

#### Cartilage explants

After 7 days of culture, cartilage explants were collected, flash-frozen in liquid nitrogen, and stored at − 70 °C. Frozen samples were homogenized with a flash-frozen mortar and pestle, suspended in 1 ml of QIAzol lysis buffer (Qiagen, Venlo, The Netherlands) and total RNA was extracted following the manufacturer’s protocol. RNA concentration and purity were assessed using a SpectraMax i3x spectrophotometer by measuring absorbance at 260 and 280 nm.

### Quantitative real-time PCR

RNA samples were treated with dsDNase (Thermo Fisher Scientific, Waltham, MA, USA) and reverse transcription was performed using the Maxima^®^ First Strand cDNA Synthesis Kit (Thermo Fisher Scientific, Waltham, MA, USA) according to the manufacturer’s instructions.

Quantitative real-time PCR (RT-qPCR) was performed using the Maxima Probe qPCR Master Mix (2X) (Thermo Fisher Scientific, Waltham, MA, USA) and a QuantStudio 1 Real-Time PCR System (Thermo Fisher Scientific, Waltham, MA, USA). The TaqMan^®^ Gene Expression Assays (Thermo Fisher Scientific, Waltham, MA, USA) were used for gene expression analysis (Table 1). All reactions were run in technical triplicates.

Thermal cycling conditions were as follows: initial denaturation at 95 °C for 10 min; 40 cycles of: Denaturation at 95 °C for 15 s; Annealing/extension at 60 °C for 60 s.

Negative controls included no-reverse transcriptase (–RT) and no-template controls (NTC). Gene expression data were normalized using the geometric mean of two reference genes: RPS9 and B2M. The fold change in gene expression was calculated with 2^− ddCt^ method.

**Table 1:** The TaqMan^®^ Gene Expression Assays used for gene expression analysis.

Gene, assay ID	Encoded protein
*RPS9*, Hs02339424_m1	Ribosomal protein S9
*B2M*, Hs00984230_m1	Beta 2 microglobulin
*COL2A1*, Hs01060345_m1	Collagen type II alpha 1 chain
*ACAN*, Hs00153936_m1	Aggrecan
*TGFBR2*, Hs00234253_m1	Transforming growth factor beta receptor 2
*MMP1*, Hs00899658_m1	Matrix metalloproteinase 1
*MMP13*, Hs00233992_m1	Matrix metalloproteinase 13
*CTSK*, Hs00166156_m1	Cathepsin K
*ESR1*, Hs01046816_m1	Estrogen receptor alpha
*PGR*, Hs01556702_m1	Progesterone receptor

### Monolayer and cartilage explant chondrocyte secretome analysis using multiplex assay

Chondrocyte proliferation medium with/without EVs after 3 days and cartilage explant supernatants with/without EVs after 3 and 7 days was used for secretome analysis using Human Cytokine/Chemokine/Growth Factor Panel Kit (Invitrogen, Thermo Fisher Scientific, Waltham, MA, USA), according to manufacturer’s instructions. The supernatants were used without dilution. The analysis was performed using a Luminex 200 instrument and a panel of 45 human cytokines/chemokines and growth factors were measured.

### Cartilage oligomeric matrix protein release in cartilage explants, immunoabsorbent assay (ELISA)

The supernatants collected from cartilage explants after treatment for 3 and 7 days were used for cartilage oligomeric matrix protein (COMP) release evaluation by ELISA (Biovendor, Brno Czech Republic) according to the manufacturer’s instructions. The medium was x500 diluted during the assay. The absorbance was measured at 450 nm using the spectrophotometer SpectraMax i3.

### Glycosaminoglycan colorimetric assay

The release of glycosaminoglycans (GAGs) was assessed in the supernatants of cartilage explants incubated with and without EVs, TGF-β3 and IL-1β for 3 and 7 days. Measurements were performed using colorimetric 1,9-dimethyl-methylene blue dye (Bicolor Life Science as-says, Carrickfergus, UK) according to the manufacturer’s instructions. The standard used for GAG calibration was provided in the kit as a bovine tracheal chondroitin 4-sulfate. The absorbance was measured at 656 nm using the spectrophotometer, SpectraMax i3.

### Histology and immunohistochemistry

For histological and IHC analysis, cartilage explants and chondrogenic pellets were collected and fixed in 10% neutral buffered formalin solution (Sigma Aldrich, Taufkirchen, Germany), then embedded into paraffin wax and cut by microtome into 4-micrometer sections. The sections were deparaffinized and stained with safranin-O and/or toluidin blue (Thermo Fisher Scientific, Waltham, MA, USA). Stained sections were analysed and evaluated by light microscopy.

The IHC staining with antibodies against collagen type II (Abcam) was performed after the antigen retrieval with a citrate buffer pH 6.0 at + 98 ◦C for 20 min and endogenous peroxidase blocking with 0.3% hydrogen peroxide for 15 min at room temperature (RT).

### Infrared absorption spectroscopy (FTIR)

Infrared spectra of the control cartilage explant and the EV-treated samples were recorded using an Alpha spectrometer (Bruker, Billerica, MA, Germany) with a room-temperature DLATGS detector and a diamond ATR accessory. Samples were pressed against the ATR crystal and measured at 4 cm^− 1^ resolution, averaging 200 scans for the sample and 500 scans for the background. Spectral intensity was normalized according to CH_2_ deformation band at 1457 cm^− 1^, and the difference spectra were constructed by subtracting control spectra from the EV-treated sample spectra. Average and standard deviation were then calculated.

### Statistical analysis

Statistical data analysis was performed using the statistical program Prism GraphPad 8.0. (Dotmatics, San Diego, CA, USA) One-way ANOVA with Tukey post hoc tests, Students t-test, Welch test are applied for group analysis (EV vs. control) and data statistical significance study, as indicated in each figure. Parametric tests (Student’s t-test, one-way ANOVA with appropriate post hoc analysis) were applied when data met assumptions of normality and homogeneity of variance, while non-parametric tests (Welch tests) were used when these assumptions were not fulfilled or when sample sizes were limited. Data considered significant when *p* ≤ 0.05. For the assessment of statistical significance of gene expression, a non-parametric Mann-Whithey test was applied with significance threshold *p* < 0,05.

## Results

### MenSC-EV characterisation according to tetraspanin expression

**Fig. 1:**
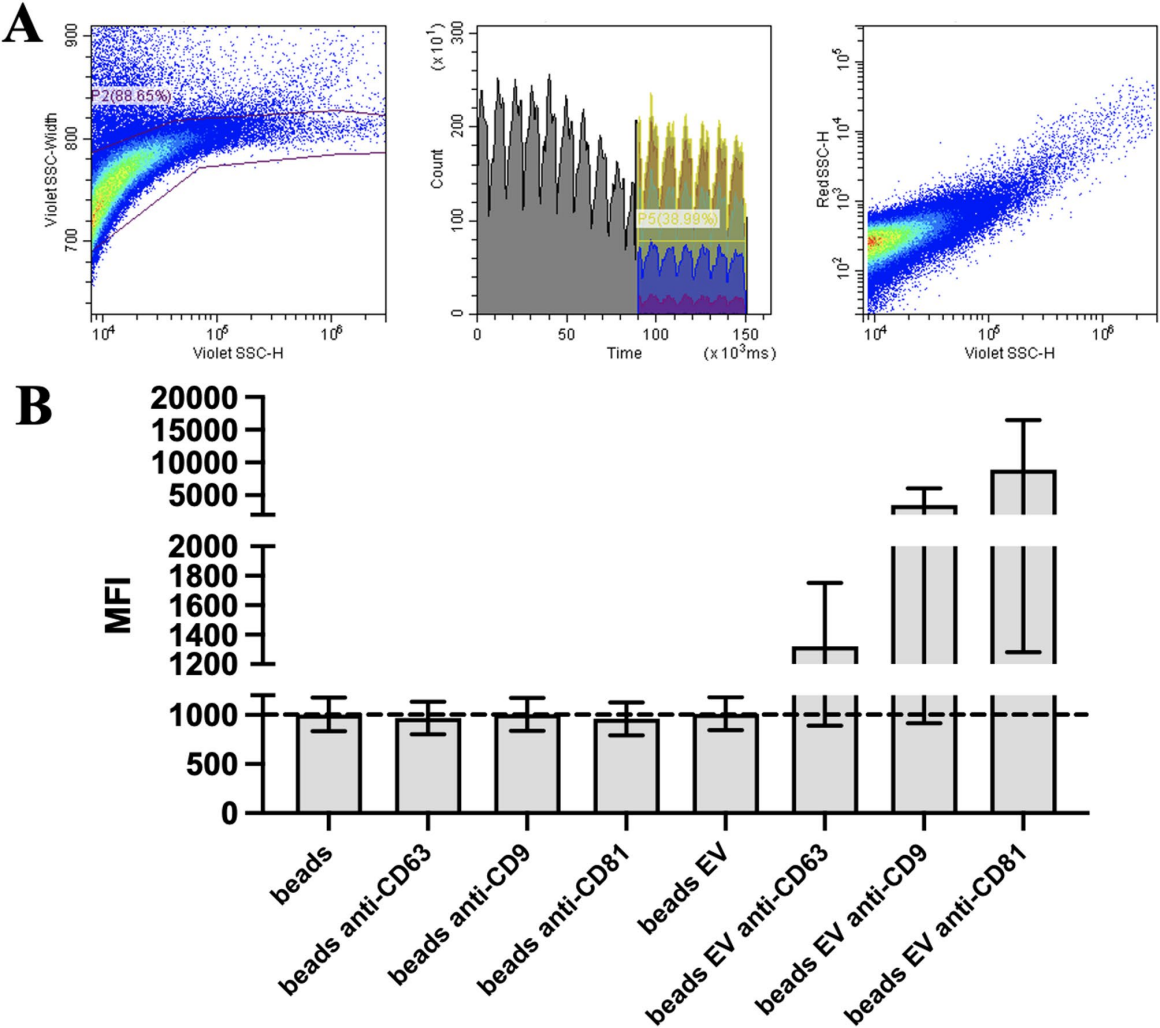
Quantification and characterisation of MenSC-EVs by the expression of tetraspanins: CD63, CD81 and CD9, using flow cytometry. **A** EV population detection according to the Apogee bead gated region (from 80 nm to 1300 nm) on the VSSC-width log x VSSC-H log cytogram to remove EV aggregates (singlet gate - P2). A rectangular gate for stable time of the collected events was set from 90 s to 150 s (P5) and a rectangular gate for the events of interest was set on the VSSC-H log x RSSC-H log cytogram containing the EV population. **B** MenSC-EV (EV) (12 mln in each position) tetraspanin expression, according to median fluorescence intensity (MFI) of phycoerythrin (PE) fluorophore combined to each antibody. Beads with EVs (Beads EV) and beads with no EVs, but combined with antibodies: anti-CD63 + PE, anti-CD9 + PE and anti-CD81 + PE were used for controls.

MenSC-EVs were successfully isolated using SEC and quantified, as confirmed by flow cytometric analysis (Fig. 1A) and further characterised according to classical tetraspanin expression. The EVs displayed a size distribution within the expected 80–300 nm range, and surface marker analysis revealed positive expression for tetraspanins CD63, CD81, and CD9, according to fluorophore PE median fluorescence intensity (MFI), confirming their EV identity (Fig. 1B).

### MenSC-EV uptake by chondrocytes

**Fig. 2:**
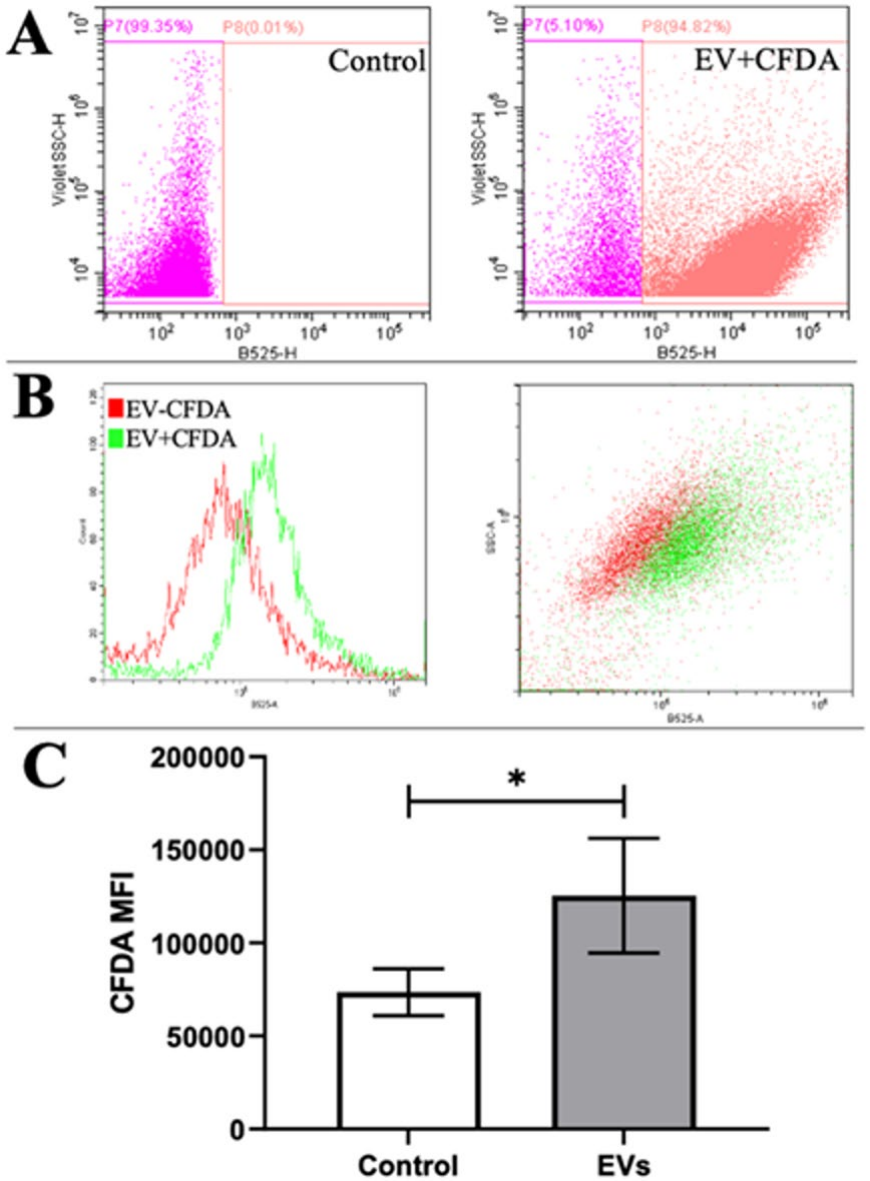
Uptake of CFDA-stained MenSC-EVs by chondrocytes and their analysis by flow cytometry. **A** CFDA-labeled MenSC-EVs (EV+CFDA) detection in B525-H channel, where control is MenSC-EVs without dye; **B** The efficiency of chondrocyte staining with stained MenSC-EVs (EV+CFDA – green colour), as compared to control (MenSC-EV without CFDA (EV-CFDA – red colour)); **C** Chondrocyte analysis after incubation with MenSC-EV (with CFDA – EVs and without - control) for 3 h, according to median fluorescence intensity (MFI) measured by flow cytometry. * Horizontal bar represent *p* ≤ 0.05 (Student’s t-test).

To determine MenSC-EVs uptake by chondrocytes, CFDA-stained EVs (EV+CFDA ± 95%), as evaluated by flow cytometry (Fig. 2A) were incubated with monolayer chondrocytes as described in methods Sect.  2.4. The flow cytometric analysis revealed efficient uptake of MenSC-EVs by chondrocytes within 3 h, as indicated by increased CFDA-positive chondrocytes (EV+CFDA) compared with controls: chondrocytes incubated with MenSC-EVs, but without CFDA staining (EV-CFDA) (Fig. 2B). The uptake efficiency was statistically significant (*P* = 0.05), according to MFI, as compared to control (EV-CFDA) (Fig. 2C).

### MenSC-EV effect on chondrocyte migration, motility speed, perimeter and proliferation

**Fig. 3:**
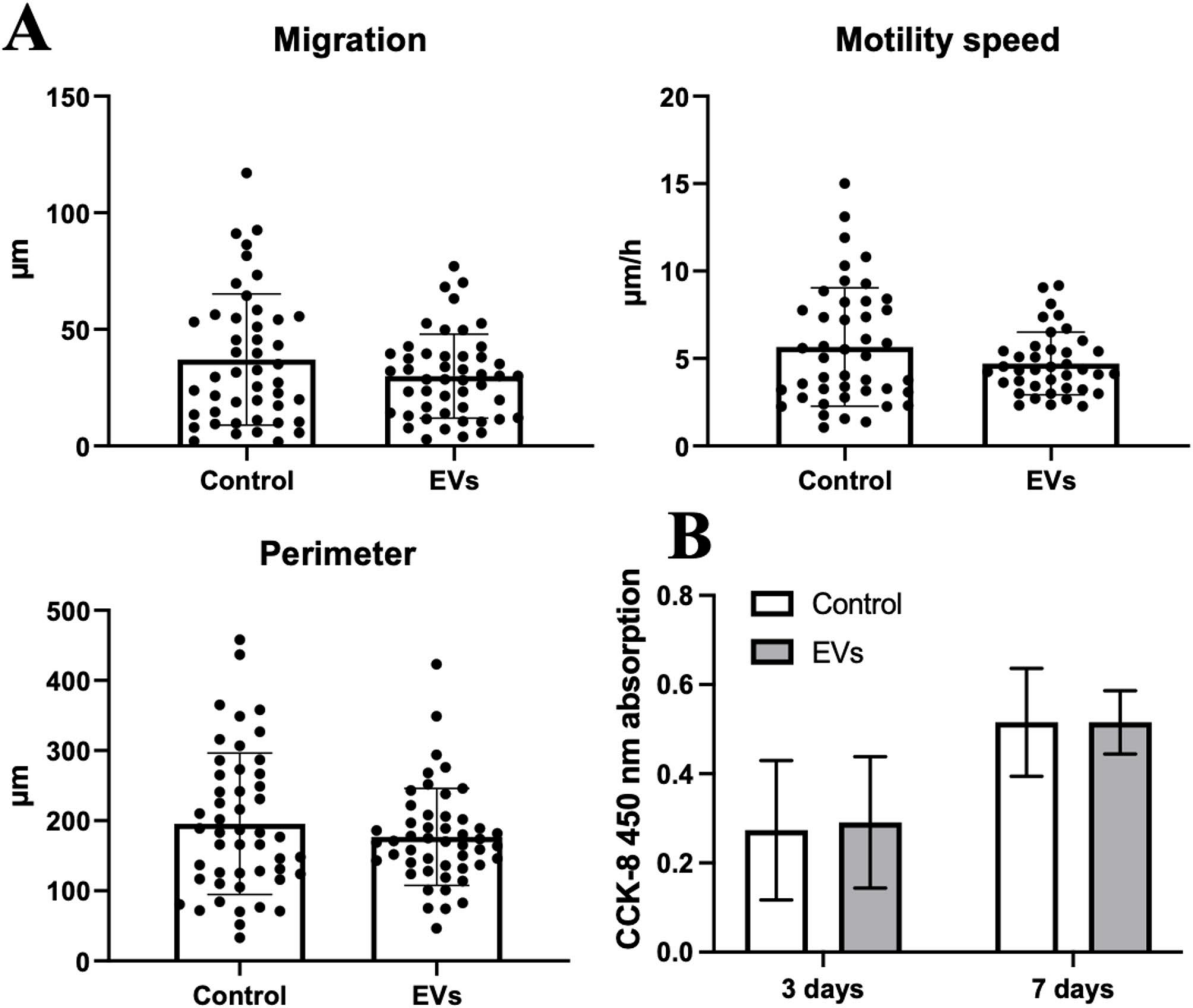
Effects of MenSC-EVs on chondrocyte proliferation, migration, motility speed and perimeter. **A** Holomonitor single-cell tracking analysis of chondrocyte migration distance, motility speed, and perimeter after 24 h of treatment with MenSC-EVs (EVs), as compared to control (without MenSC-EVs) (holomonitor). Each dot represents a single donor’s result. **B** Chondrocyte proliferation analysis after treatment with MenSC-EVs for 3 days, after 3 and 7 days, CCK-8 assay, measured with spectrophotometer.

In order to further analyse the effect of MenSC-EVs on chondrocyte functions, we firstly evaluated chondrocyte migration, motility speed, perimeter and proliferation capacity. Holomonitor single-cell tracking demonstrated that MenSC-EVs preserved normal chondrocyte morphology, migration, motility speed and perimeter, without affecting any cellular properties in vitro (Figs. 3A). The holomonitor system presents migration as a distance that a cell travels from its original position, reflecting directional movement, whereas motility speed describes the total distance covered by a cell in an hour, including all changes in direction. Thereby, the system characterises the overall activity of the cell movement. Quantitative tracking analysis showed that both migration distance and motility speed were comparable between control and MenSC-EV treated groups, indicating that MenSC-EVs did not affect both functional parameters of chondrocytes. Similarly, cell perimeter measurements remained stable across groups. Therefore, 100EV/cell did not induce morphological changes in chondrocytes. In addition, cell proliferation assay has shown that chondrocytes remained viable and metabolically active upon MenSC-EV treatment, with comparable proliferation rates to controls up to 7 days (Fig. 3B).

### MenSC-EV effect on chondrocyte sex hormone receptor levels

**Fig. 4:**
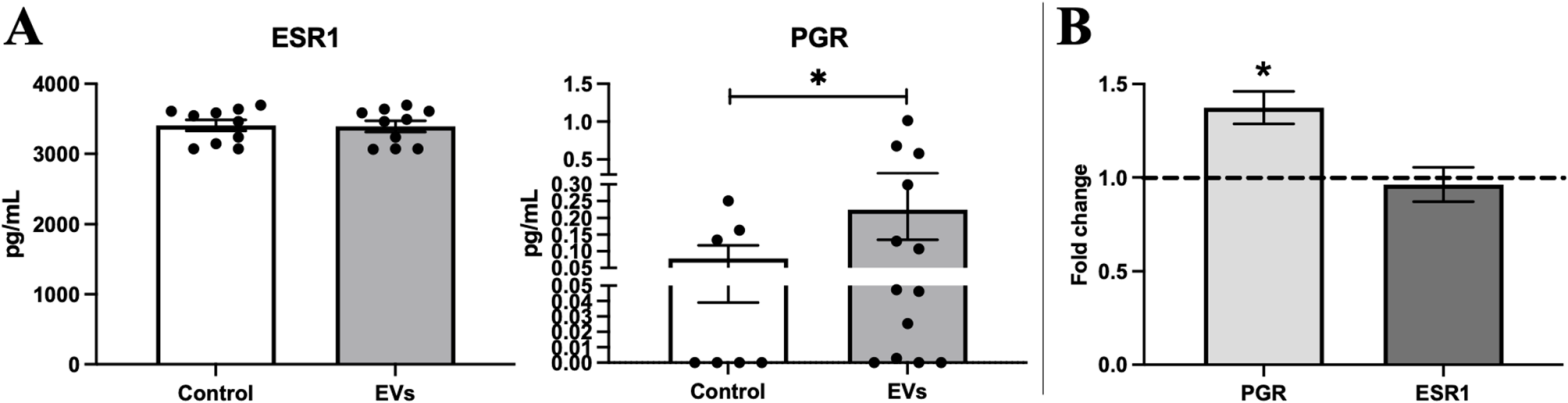
MenSC-EV effect on chondrocyte sex hormone receptor protein and gene expression. **A** Estrogen receptor 1 alpha (ESR1) and progesterone receptor (PGR) protein synthesis in chondrocyte monolayer after stimulation with MenSC-EVs for 3 days (ELISA). Control – chondrocytes cultured under the same conditions, but without MenSC-EVs. Each dot represents a single donor’s result. **B** ESR1 and PGR gene expression in chondrocyte monolayer, after stimulation with/without MenSC-EVs for 3 days. Relative mRNA level presented after normalization to two housekeeping genes (B2M and RPS9). Fold change on y axys represent EV/control ratio. * and horizontal bar represent *p* ≤ 0.05 (Welch non-parametric test).

Chondrocyte sex hormone receptors – ESR1 and PGR were analysed by protein and gene expression in chondrocyte monolayer, after treatment with MenSC-EVs for 3 days. EV treatment did not affect protein levels of ESR1, while significantly increased PGR in chondrocyte lysates (*P* = 0.02), as assessed by ELISA (Fig. 4A). Gene expression analysis further confirmed upregulation of PGR mRNA in EV-treated cells (*P* = 0.04), while ESR1 remained unchanged, as compared to non-stimulated with EVs chondrocytes - controls (Fig. 4B).

### MenSC-EV effect on chondrocyte secretome in monolayer cultures

**Fig. 5:**
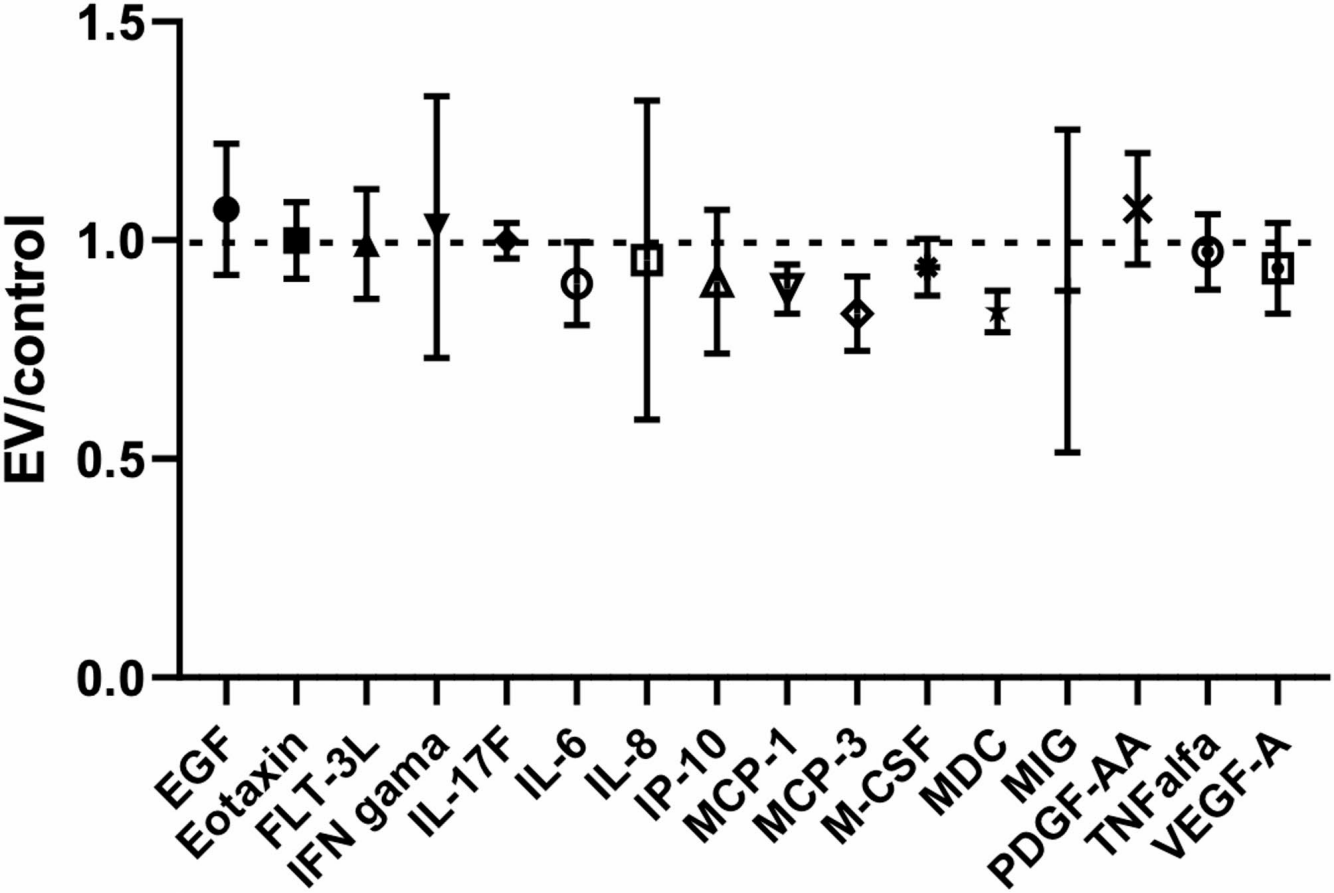
MenSC-EV effects on chondrocyte secretome, after treating with EVs for 3 days, analysed by Luminex. Control–chondrocyte secretome without treatment with MenSC-EVs. EV ratio to control is presented.

To further evaluate the effects of MenSC-EVs on chondrocyte secretory activity, we performed Multiplex analysis of chondrocyte culture supernatants. Among the whole panel of 45 analysed factors, endothelial growth factor (EGF), eotaxin, FMS-like tyrosine kinase 3 ligand (FLT-3 L), interferon gamma (IFN gamma), interleukin 17 F, 6 and 8 (IL-17 F, IL-6, IL-8), IFN gamma-induced protein 10 (IP-10), Monocyte chemoattractant protein 1 and 3 (MCP-1, MCP-3) macrophage colony-stimulating factor (M-CSF), macrophage derived chemokine (MDC), monokine induced by gamma Interferon (MIG), platelet-derived growth factor AA (PDGF-AA), tumor necrosis factor alpha (TNFalfa), vascular endothelial growth factor A (VEGF-A) were secreted in chondrocyte cultures. The results demonstrated that treatment with MenSC-EVs did not induce alterations in cytokine/growth factor secretion and chondrocytes maintained a largely stable cytokine secretion profile, without any significant change to the treatment with MenSC-EVs (Fig. 5).

### Chondrocyte ECM synthesis after treatment with MenSC-EVs

**Fig. 6:**
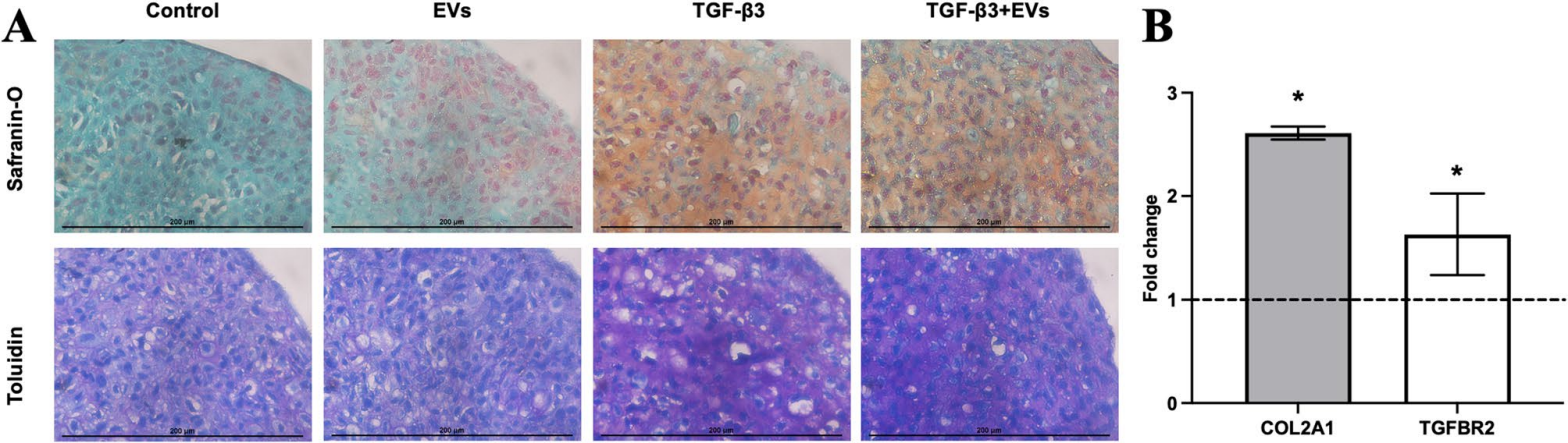
MenSC-EV effect on chondrocytes in 3D pellets, after 3 days of treatment and chondrogenic induction by MenSC-EVs in a chondrogenic medium containing TGF-β3 (10 ng/mL) for 21 days. **A** Histological evaluation of the chondrogenic pellets stained with safranin-O and toluidin blue, x400; **B** RT-qPCR of TGFBR2 and COL2A1 gene expression in MenSC-EV with TGF-β3-treated pellets compared to TGF-β3 alone. Relative mRNA level presented after normalization to two housekeeping genes (B2M and RPS9). Fold change on y axis represent TGF-β3 + EV/TGF-β3 ratio. * represent *p* ≤ 0.05 (one-way ANOVA test).

Chondrocyte capacity to rebuild cartilage ECM was assessed in chondrogenic pellet models cultured with chondrogenic medium, as discussed in the methods Sect.  2.7. Chondrocyte pellets cultured in chondrogenic medium with TGF-β3 and TGF-β3 + EVs showed increased ECM synthesis, according to histological pellet staining with classical cartilage ECM staining dyes: safranin O and toluidin blue, as compared to non-treated with MenSC-EVs control (Fig. 6A). Moreover, it was noticed that MenSC-EV-only stimulated pellets possessed higher proteoglycan content, as compared to non-treated control, due to safranin O staining. Addition of TGF-β3 increased the production of ECM and COL2A1 gene expression, while the additional treatment with MenSC-EVs significantly enhanced COL2A1 (*P* = 0.04) and TGF-β3 receptor (TGFBR2) (*P* = 0.009) gene expression, as compared to TGF-β3 alone, (Fig. 6B). Chondrogenic medium only and MenSC-EV treatment without addition of TGF-β3 did not affect gene expression (results are not presented).

### MenSC-EV effects on cartilage explants under inflammatory and regenerative conditions

**Fig. 7:**
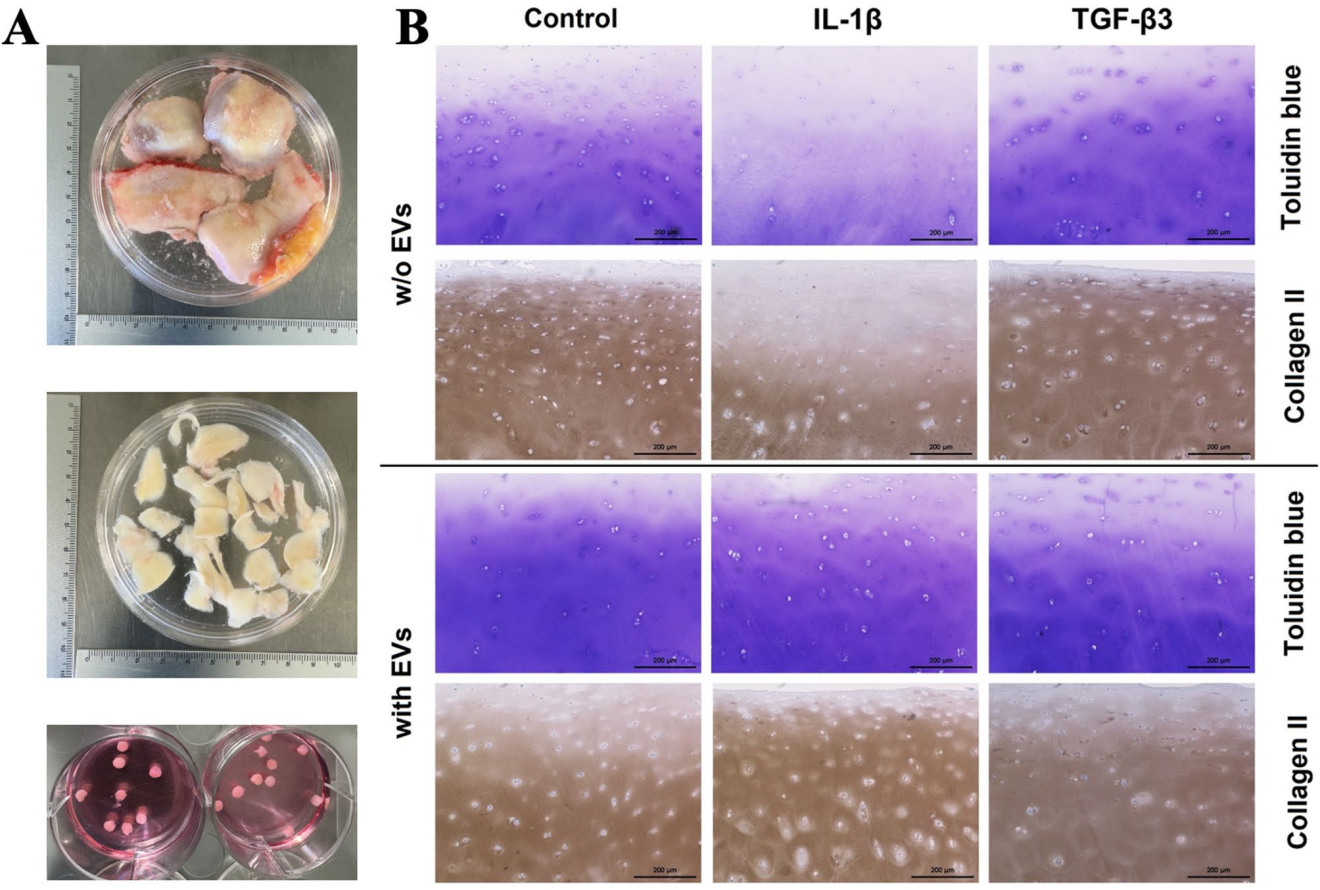
Immunohistochemical/histological sample analysis of cartilage explants, cultured with/without IL-1β (10 ng/mL), TGF-β3 (10 ng/mL) for 7 days and MenSC-EVs for 3 days. **A** Macroscopic image of cartilage samples received from the hospital and its processing to isolating explants; **B** Histological and IHC staining of cartilage explants with anti-collagen II antibodies and toludin after 7 days). x100 magnification.

After assessing the effects of MenSC-EVs on monolayer chondrocyte cultures, we next examined cartilage explants as a native, intact cartilage model (Fig. 7A).

To evaluate cartilage responses under conditions mimicking the in vivo environment, cartilage explants were isolated and cultured with IL-1β to model inflammation, or with TGF-β3 as a regenerative factor promoting ECM synthesis. MenSC-EV treatment was applied as an additional factor under all conditions.

Figure 7B presents histological and IHC staining of cartilage explants after 7 days of culture. Toluidine blue staining indicated a slight increase in cartilage ECM synthesis in explants treated with MenSC-EVs alone compared to untreated controls. However, IHC labeling for collagen type II did not show detectable differences between these same groups.

As expected, IL-1β stimulation induced pronounced cartilage ECM degradation, as evidenced by reduced toluidine blue and collagen type II staining. Notably, the addition of MenSC-EVs to IL-1β-stimulated explants markedly attenuated ECM loss, maintaining strong staining for both toluidine blue and collagen type II. Explants treated with TGF-β3 alone, or with TGF-β3 in combination with MenSC-EVs, displayed robust toluidine blue and collagen type II staining, with no visible differences between the two conditions.

**Fig. 8:**
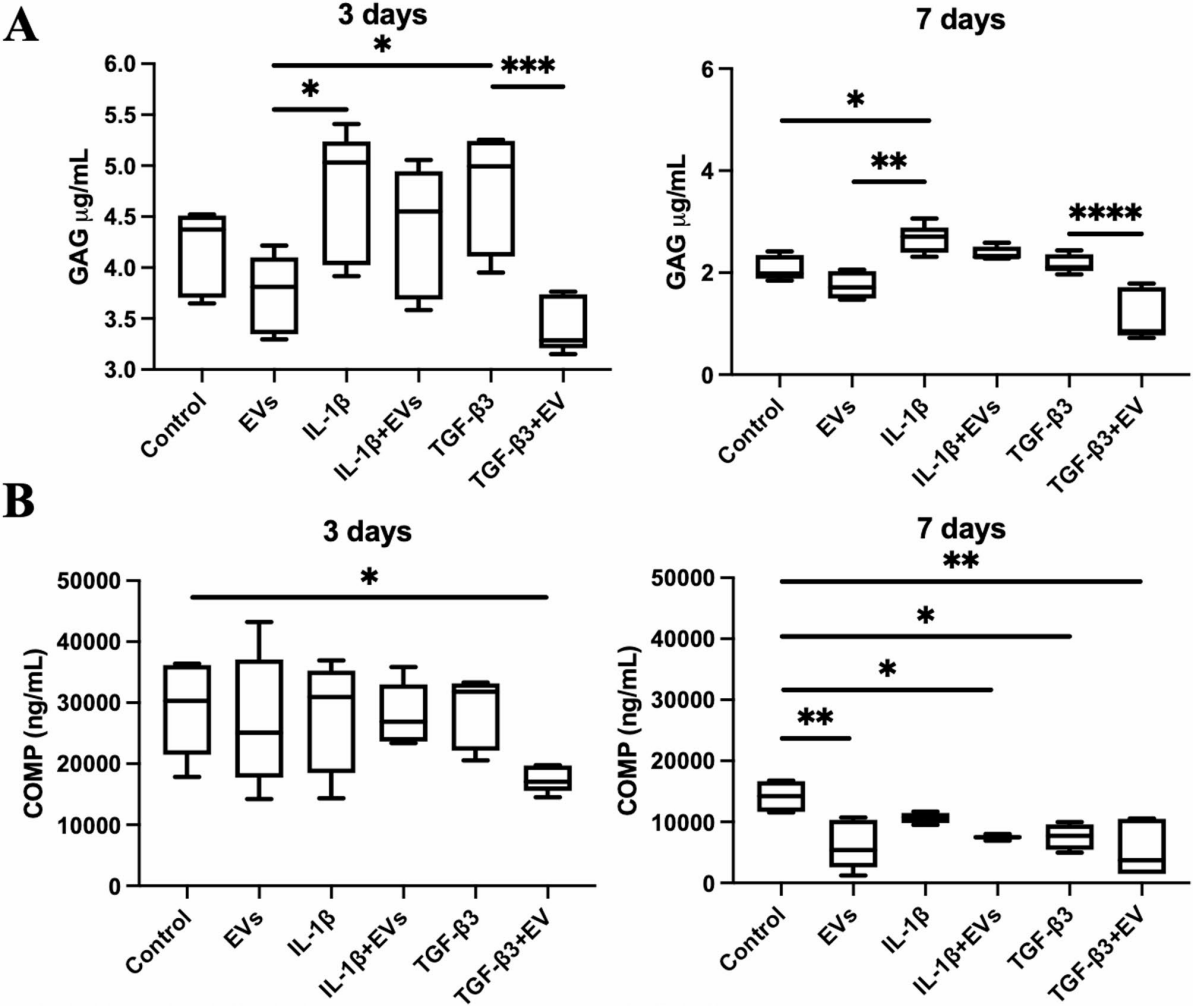
GAG release assay (**A**) and COMP (ELISA) (**B**) in cartilage explants after treatment with MenSC-EVs for 3 days, as well as with/without IL-1β (10 ng/mL), TGF-β3 (10 ng/mL) after 3 and 7 days. Control – cartilage explants without treatment with MenSC-EVs. *P ≤ 0.05, ** *P* ≤ 0.01, *** *P* ≤ 0.001, **** *P* ≤ 0.0001 (one-way ANOVA test).

Moreover, cartilage explant medium was analysed by measuring released GAGs and COMP after 3 and 7 days in all of the samples (Fig. 8). IL-1β exhibited remarkable matrix degradation, characterised by increased GAG release after 7 days (*P* = 0.012). Treatment with MenSC-EV for 3 days reduced both GAG and COMP release after 3 and 7 days in all of the treatment groups, while significant results were obtained in GAG release after 3 days comparing MenSC-EV treatment with IL-1β (*P* = 0.014) and TGF-β3 (*P* = 0.015) only treated samples, also TGF-β3 and TGF-β3 + EV groups (*P* = 0.0006); COMP release after 3 days in control and TGF-β3 + EV group (*P* = 0.049); GAG release after 7 days in MenSC-EV treatment with IL-1β (*P* = 0.007) and TGF-β3 and TGF-β3 + EV groups (*P* = 0.0001); COMP release after 7 days in MenSC-EVs only group compared to control (*P* = 0.0028), IL-1β + EVs group compared to control (*P* = 0.0184) and TGF-β3 + EVs group compared to control (*P* = 0.001).

### MenSC-EV effects on chondrocyte secretome in cartilage explants

**Fig. 9:**
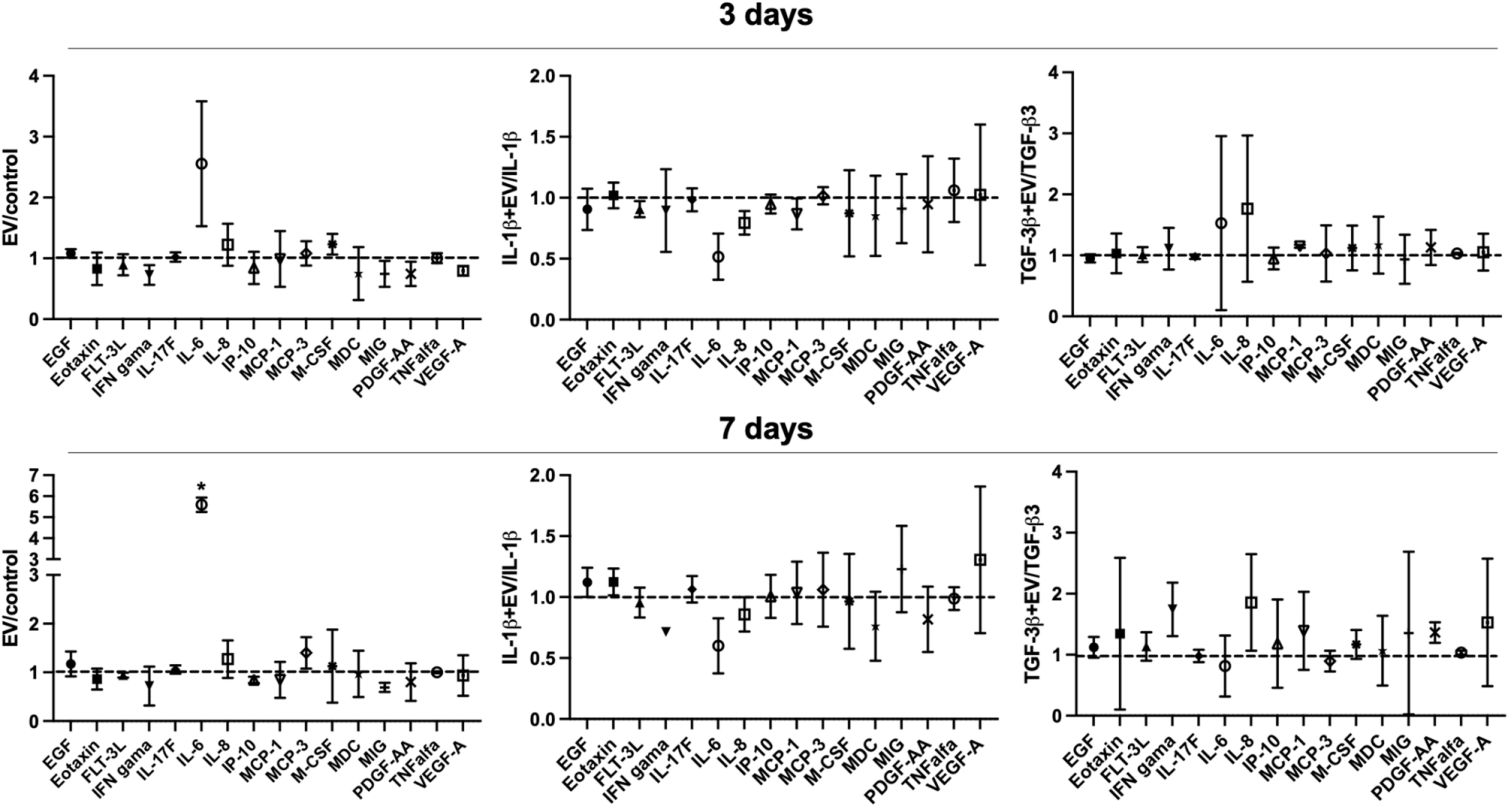
MenSC-EV effects on chondrocyte secretome in cartilage explants, after treating with EVs for 3 days, as well as with/without IL-1β (10 ng/mL), TGF-β3 (10 ng/mL) after 3 and 7 days, analysed by Luminex. Control – chondrocyte secretome without treatment with MenSC-EVs. The graphs present MenSC-EV ratio to control, also MenSC-EV + IL-1β ratio to IL-1β treatment alone, and MenSC-EV + TGF-β3 ratio to TGF-β3 treatment alone are presented. * represents *p* ≤ 0.05 (one-way ANOVA test).

Cartilage explant cytokine/growth factor analysis was evaluated after 3 and 7 days in culture, after treatment with MenSC-EVs for 3 days. Multiplex analysis of cartilage explant supernatants showed that MenSC-EV treatment maintained a balanced cytokine and growth factor profile under both regenerative and inflammatory conditions (Fig. 9). Among the whole panel of 45 analysed factors, endothelial growth factor (EGF), eotaxin, FLT-3 L, interferon gama (IFN gama), IL-17 F, IL-6, IL-8, IP-10, MCP-1, MCP-3, M-CSF, MDC, MIG, PDGF-AA, TNFalfa, VEGF-A were secreted in explant cultures, however, without any significant change to the treatment with MenSC-EVs, except for IL-6 after 7 days of culture (*P* = 0.0043).

### Gene expression in cartilage explants treated with MenSC-EVs

**Fig. 10:**
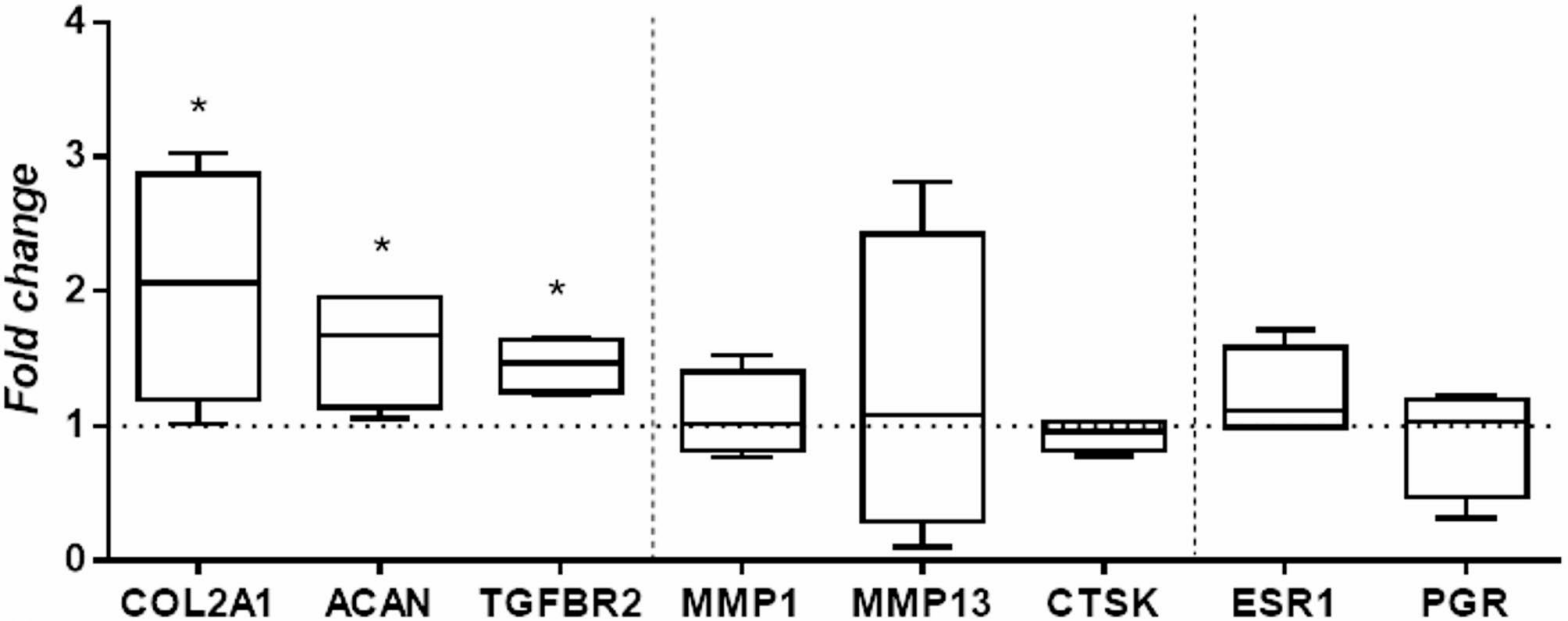
Gene expression analysis (RT-qPCR) of cartilage explants, after treatment with MenSC-EVs. Collagen type II (COL2A1), aggrecan (ACAN), transforming growth factor beta receptor 2 (TGFBR2), metalloproteinase 1 (MMP1), metalloproteinase 13 (MMP13), cathepsin K (CTSK), estrogen receptor 1 alpha (ESR1) and progesterone (PGR) receptor genes were evaluated in explants after 7 days in culture. Relative mRNA level presented after normalization to two housekeeping genes (B2M and RPS9). Fold change on y axis represents EV/Control ratio. * represents *p* ≤ 0.05 (non-parametric Mann Whithey test).

After analysing cartilage explant cultures under inflammatory and regenerative conditions, we evaluated gene expression analysis after treatment with MenSC-EVs. RT-qPCR analysis of cartilage explants was performed after 7 days in culture and revealed significant up-regulation of cartilage-specific genes COL2A1, ACAN and TGFBR2 (*P* = 0.029) expression in cartilage explants treated with MenSC-EVs, as compared to controls (Fig. 10). These results suggest that EVs can stimulate the production of hyaline cartilage-specific ECM components. On the other hand, there was no change in the expression of matrix-degrading enzymes’ genes – MMP1, MMP13 and CTSK and sex hormone receptors-related genes ESR1 and PGR.

### 10. Infrared absorption spectroscopy of cartilage explants treated with MenSC-EVs

**Fig. 11:**
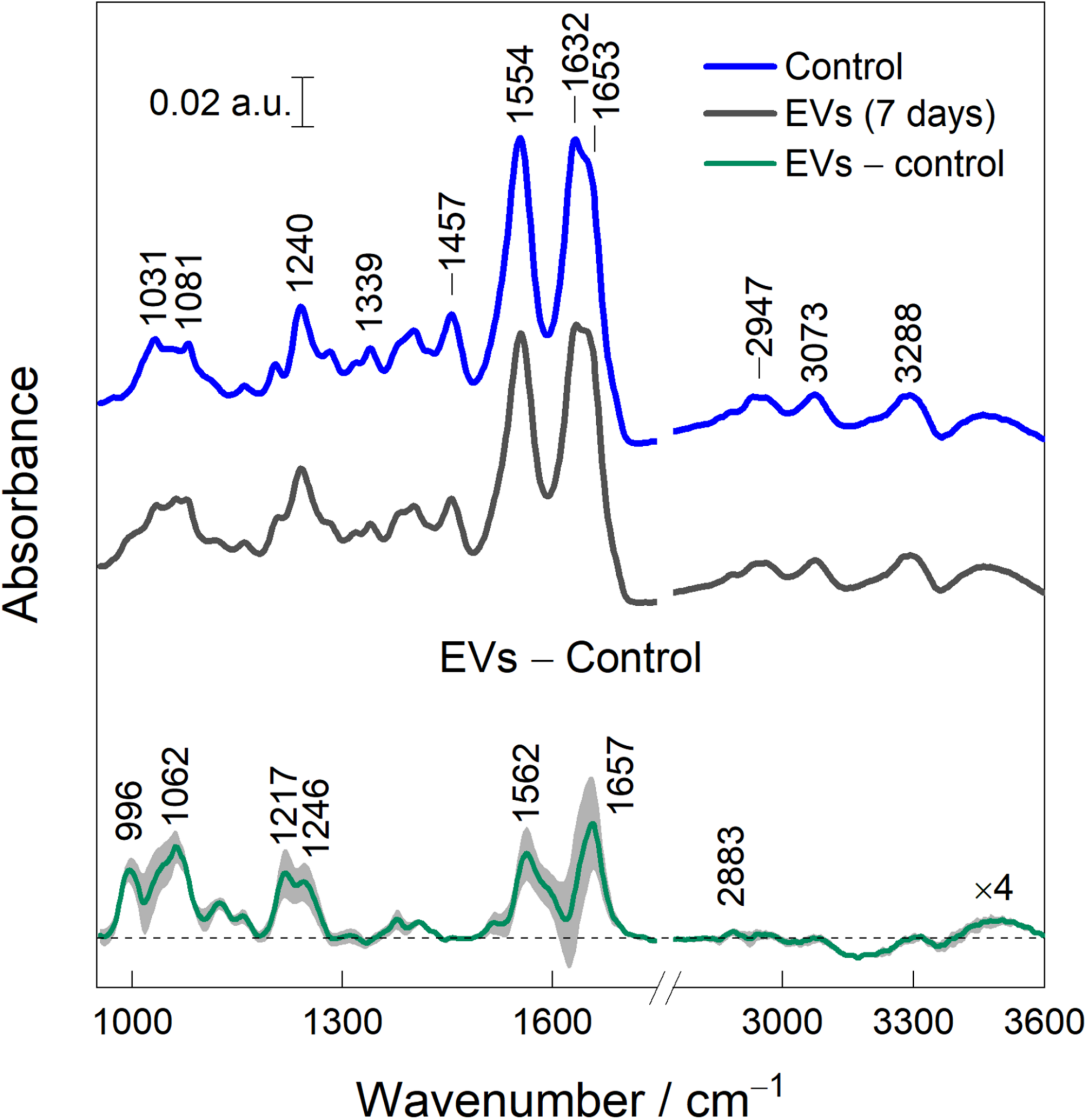
FTIR spectra of cartilage explants cultured with (EVs) or without MenSC-EVs (control) for 7 days. The difference spectrum is constructed by subtracting control spectra from EVs. Spectra are normalized according to the CH_2_ deformation at 1457 cm^− 1^. Average and standard deviation (grey area) are calculated from three independent samples.

FTIR spectra provide label-free molecular vibrational signature of cartilage explants cultured for 7 days with or without MenSC-EVs are shown in Fig. 11. Spectra were dominated by amide I (1600–1700 cm^− 1^) and amide II (1554 cm^− 1^) from proteins, together with CH_2_ bending near 1457 cm^− 1^^23,24^. The amide I exhibited a maximum at 1632 cm^− 1^, consistent with b-sheet-rich structures and a shoulder near 1653 cm^− 1^ attributed to a-helix or disordered protein conformations^23^. Band near 1030 cm^− 1^ originated from C–O stretch and C–OH bend from proteoglycans and carbohydrates, notably, glycogen, while peaks at 1081 and 1240 cm^− 1^ corresponded to vibrations of phosphate groups from lipids^25,26^. In the high frequency region, modes at 2947, 3073, and 3288 cm^− 1^ were assigned to CH_3_ asymmetric stretching, amide B, and amide A, respectively (Kong, et al., 2007). The difference spectrum (EV-treated minus control) revealed changes primarily in the amide I/II region, indicating EV-dependent alterations in the protein matrix. The 1657 cm^− 1^ position is linked with the a-helix structure of the collagen II produced in the ECM, and the mode at 1062 cm^− 1^ is linked with proteoglycans.

## Discussion

In this study, we demonstrated that MenSC-EVs have the potential to stimulate chondrocytes to synthesize ECM, highlighting their possible application as a novel, cell-free therapeutic strategy for cartilage repair. While MSC-derived EVs from other sources, such as bone marrow or adipose tissue, have been studied extensively on cartilage tissue^8,10,27^, MenSC-EVs remain relatively unexplored. Our findings, therefore, expand the knowledge of EV-based cartilage regeneration strategies, particularly relevant for female-specific regenerative medicine approaches and are supported by recent reviews emphasizing the promise of MSC-derived EVs for cartilage regeneration, though direct MenSC data have been lacking^28,29^.

We first confirmed successful EV isolation using SEC and validated the vesicles by detecting the expression of the canonical tetraspanins CD63, CD81, and CD9 (Fig. 1), which are widely used to detect isolated EVs in different fluids/tissues^8,30,31^. We optimized the EV concentration prior to all experiments using a dose–response approach in monolayer chondrocyte cultures (data not shown), testing 50, 100, and 500 EVs per cell. At the lowest dose (50 EVs/cell), CFDA-labeled EV uptake by chondrocytes was minimal, indicating insufficient vesicle–cell interaction. In contrast, exposure to a high EV load (500 EVs/cell) resulted in excessive vesicle internalization and was associated with impaired chondrocyte migratory capacity, suggesting cellular overload and potential functional disturbance. Based on these observations, a dose of 100 EVs per cell was selected as an optimal compromise, ensuring efficient EV uptake while preserving normal chondrocyte morphology, viability, and migratory behavior. This concentration was therefore used consistently throughout all monolayer, 3D pellet, and cartilage explant experiments to enhance experimental reproducibility. MenSC-EV-CFDA uptake experiments demonstrated efficient internalization of MenSC-EVs by chondrocytes already after 3 h (Fig. 2), consistent with previous studies showing rapid incorporation of MSC-EVs into recipient cells^10^. Importantly, despite efficient uptake, MenSC-EVs did not significantly influence chondrocyte proliferation, migration, perimeter or motility speed (Fig. 3). These findings demonstrate that MenSC-EVs support the maintenance of normal chondrocyte morphology, viability and dynamic cellular properties in vitro. Chen et al. (2022) reported that EVs derived from Wharton’s jelly MSCs promote chondrocyte proliferation and migration^32^. This discrepancy may reflect intrinsic source-related differences, as MenSC-EVs carry a unique secretome profile influenced by cyclical hormonal environment, which could favor matrix regulation over proliferation. Our previous findings show that MenSC paracrine factors in conditioned media stimulate chondrogenic differentiation of bone marrow MSCs (BMMSCs) through secretion of TGF-β3 and activin A, and is able to preserve cartilage matrix by reducing degradation, partly via control of GAG release and activin A pathway signaling^22,29^. A particularly interesting observation was the upregulation of PGR expression in chondrocytes after MenSC-EV treatment, while ESR1 receptor expression remained unaffected (Fig. 4). This may be clinically significant, as sex hormone receptors in cartilage have been associated with post-menopausal OA pathogenesis^2,18^. The ability of MenSC-EVs to modulate PGR levels may thus represent a previously unrecognized mechanism contributing to female-specific cartilage homeostasis and repair. These data complement recent studies indicating that MenSCs contribute to chondrogenic differentiation and protect cartilage matrix via paracrine secretion of key growth factors^22^. The observed upregulation of PGR in monolayer chondrocytes following MenSC-EV treatment is interpreted as a modulation of chondrocyte receptor expression rather than a direct hormonal or hormone-like therapeutic effect. However, neither PGR nor ESR1 gene expression were upregulated in cartilage explants after treatment with MenSC-EVs (Fig. 10). This discrepancy could be explained by the fact that cartilage explants, unlike monolayer chondrocyte cultures, represent a more complex tissue environment where ECM density, limited EV penetration, and reduced cellular uptake efficiency may restrict the transcriptional response to MenSC-EV treatment. These aspects should be carefully studied in the future by modulating EV concentration, uptake dynamics, and culture conditions in cartilage explants.

Importantly, multiplex cytokine revealed that MenSC-EV treatment did not induce a pro-inflammatory response in chondrocytes or cartilage explants, with the exception of IL-6 changes (Figs. 5 and 9). IL-6 is a pleiotropic cytokine, capable of exerting both pro- and anti-inflammatory effects depending on the signaling context^33,34^. The observed modulation of IL-6 may not necessarily represent a detrimental response but could instead reflect a complex immunoregulatory adjustment, potentially even contributing to tissue protection. This interpretation is consistent with the concept that MenSC-EVs, like EVs from other MSC sources, exert anti-inflammatory or inflammation-modulating effects^11,35^. Although we did not observe major pro-inflammatory responses, EV effects may depend on dose, donor age, and preconditioning. The absence of strong inflammatory signaling is a crucial advantage for therapeutic applications, where immunomodulatory safety is essential. In accordance with these results, previous studies also demonstrate MenSC immunomodulatory responses, for instance in recent research demonstrates MenSCs can enrich pro-regenerative miRNAs following TNF-α preconditioning, further reducing inflammatory signaling and supporting matrix homeostasis^36^. Similarly, BMMSC-EVs can reverse IL-1β-induced downregulation of key cartilage ECM genes (COL2A1, ACAN) and suppress catabolic factors in OA chondrocytes, suggesting that EVs exert beneficial effects even under inflammatory stress^37^, which is in agreement with the data of the present study.

Functional assays provided further evidence of MenSC-EV regenerative potential. In chondrocyte pellet cultures, MenSC-EVs enhanced ECM deposition and significantly upregulated expression of cartilage-specific genes such as collagen type II and TGF-β3 receptor (Fig. 6). These findings are in line with previous reports of BMMSC or adipose tissue-derived MSC EVs stimulating chondrogenic gene expression^8,10^, but this is the first demonstration using MenSC-EVs. The systematic review by Piñeiro-Ramil et al. (2025) summarizes multiple works showing that MSC small EVs improve ECM composition, reduce chondrocyte apoptosis, and inhibit inflammation, reinforcing our conclusion that the regenerative effect observed with MenSC-EVs is part of a broader, reproducible phenomenon among different MSC sources^38^. Similarly, in cartilage explant models, EV treatment increased ECM density and reduced the release of degradation markers COMP and GAGs (Figs. 7 and 8), even under inflammatory stimulation with IL-1β. These protective effects were further supported by upregulation of collagen II, aggrecan, and TGF-β receptor gene expression (Fig. 10), suggesting that MenSC-EVs can both preserve and restore cartilage matrix integrity under degenerative conditions by an increase of TGF-β receptors in chondrocytes. Interestingly, MMP13 gene expression was not altered by MenSC-EV treatment, which may indicate that the observed ECM preservation is not mediated by direct suppression of catabolic enzymes but rather through enhanced anabolic signaling. This indirect mechanism is consistent with the concept of EVs acting as paracrine mediators that restore tissue homeostasis rather than exerting a single targeted effect.

FTIR spectroscopy provided complementary, label-free confirmation of MenSC-EV–associated ECM preservation in cartilage explants (Fig. 11). Spectra normalized to 1457 cm^− 1^ mode showed EV-dependent changes primarily within amide I/II, with features at ~ 1657 cm^− 1^ attributable to α-helical collagen II and at ~ 1062 cm^− 1^ assigned to proteoglycans. This reflect changes in the protein component of the matrix, which is consistent with enhanced collagen content, particularly collagen type II. In parallel, the increased intensity of carbohydrate-associated bands corresponds to higher proteoglycan levels. These spectroscopic findings align with the histological and IHC detection of collagen II and proteoglycan-rich ECM, as well as with the upregulation of cartilage-specific genes (COL2A1, ACAN, and TGFBR2). Together, these results indicate that FTIR captures molecular-level changes in cartilage composition that support and independently validate the tissue- and gene-level evidence of MenSC-EV–mediated matrix restoration. Previous studies also demonstrate beneficial EV effects on cartilage gene expression and ECM synthesis. For instance, hypoxia-preconditioned MSC-EVs enriched in miR-122-5p augmented chondrocyte regeneration, likely via autophagy pathways^39^.

Safety and translational feasibility are critical considerations for EV-based therapies. Compared with cell transplantation, EVs carry a lower risk of immunogenicity, tumorigenicity, and vascular occlusion, while still retaining the paracrine bioactivity of their parent cells^31,36,40,41^. MenSCs, in particular, represent an attractive source: they can be collected repeatedly and non-invasively from healthy donors, avoiding the morbidity and ethical concerns associated with bone marrow or adipose tissue harvest^14,35^. Importantly, MenSC-EVs inherit the immunomodulatory and pro-regenerative profile of MenSCs, and our data suggest that they promote cartilage redifferentiation and matrix protection without triggering inflammatory responses. From a clinical perspective, this offers two key advantages. First, MenSC-EVs could fill a therapeutic gap in OA, where conventional MSC therapies are limited by donor variability, invasive harvest, and safety concerns. Second, their hormone-receptor–modulating properties may be particularly relevant for female patients, who are disproportionately affected by post-menopausal OA. These features position MenSC-EVs as a distinctive, sex-specific, and ethically accessible cell-free therapy.

Taken together, our findings establish MenSC-EVs as promising mediators of cartilage regeneration, capable of stimulating ECM synthesis without triggering inflammation. Given the accessibility of menstrual blood as a cell source, MenSCs represent a highly feasible and ethically non-invasive option for generating regenerative EVs. However, further studies are needed to determine the specific cargo (miRNAs, proteins, lipids) responsible for the observed effects, and to validate the therapeutic efficacy of MenSC-EVs in preclinical animal models of OA, or post-traumatic cartilage injury.

### Limitations and future directions

Although this study provides novel insights, several limitations must be acknowledged. First, our findings are based on in vitro and ex vivo OA chondrocyte and cartilage explant models, and in vivo validation is needed to determine MenSC-EV biodistribution, stability, and regenerative potential under physiological conditions. Second, the long-term efficacy of MenSC-EV treatment remains unknown, as our experiments focused on short-term outcomes. Finally, donor-related factors must be considered: MenSCs are derived from reproductive-age women, whereas OA patients are typically older, and male donors are inherently excluded. Future studies should therefore include in vivo models to evaluate long-term efficacy and address donor-related considerations to support clinical translation.

## **Conclusions**

This work is the first to demonstrate that MenSC-EVs stimulate chondrocyte ECM synthesis and cartilage preservation under both normal and inflammatory conditions. Histological analysis, infrared spectroscopy, and gene expression profiling confirmed the safety and protective effects of MenSC-EVs, establishing their capacity to promote cartilage matrix production.

These results position MenSC-EVs as a promising cell-free therapeutic candidate with key translational advantages: non-invasive collection from a renewable source – menstrual blood, reduced immunogenicity compared to cell-based therapies, and sex-specific relevance for female patients. The ability to derive EVs repeatedly through an ethically acceptable, minimally invasive procedure addresses limitations of conventional MSC sources.

Future studies should elucidate the specific molecular cargo (miRNAs, proteins, lipids) responsible for regenerative effects, validate therapeutic efficacy in preclinical OA models, and investigate donor-related variability to optimize and advance clinical translation.

## Data Availability

All data generated or analysed during this study are included in this published article. The raw datasets used and/or analysed during the current study are available from the corresponding author on reasonable request.

## Abbreviations

ACAN: Aggrecan gene
BSA: Bovine serum albumin
BMMSCs: Bone marrow–derived mesenchymal stromal cells
CCK-8: Cell Counting Kit–8
cDNA: Complementary DNA
COL2A1: Collagen type II alpha 1 chain
COMP: Cartilage oligomeric matrix protein
CTSK: Cathepsin K
DMEM: Dulbecco’s Modified Eagle Medium
DPBS: Dulbecco’s phosphate–buffered saline
ECM: Extracellular matrix
EGF: Endothelial growth factor
ELISA: Enzyme–linked immunosorbent assay
ERα/ERβ (ESR1/ESR2): Estrogen receptor alpha/beta
EVs: Extracellular vesicles
FBS: Fetal bovine serum
FLT-3L: FMS–like tyrosine kinase 3 ligand
GAG: Glycosaminoglycan
IHC: Immunohistochemistry
IL-1β: Interleukin–1 beta
IL-17F: nterleukin–17 F
IL-6: Interleukin–6
IL-8: Interleukin–8
INF gamma: Interferon gamma
IP-10: IFN gamma–induced protein 10
ITS: Insulin–transferrin–selenium
M-CSF: Macrophage colony–stimulating factor
MCP1; MCP–3: Monocyte chemoattractant protein 1 and 3
MDC: Macrophage derived chemokine
MenSCs: Menstrual blood–derived mesenchymal stromal cells
MFI: Median fluorescence intensity
MIG: Monokine induced by gamma interferon gamma
MMP1/13: Metalloproteinase 1/13
mRNA: Messenger RNA
MSC(s): Mesenchymal stromal cell(s)
OA: Osteoarthritis
PBS: Phosphate–buffered saline
PDGF: AA–Platelet–derived growth factor AA
PGR: Progesterone receptor
PE: Phycoerythrin
qPCR (RT-qPCR): Quantitative real–time polymerase chain reaction
SEC: size exclusion chromatography
TGF-β3: Transforming growth factor beta 3
TGFBR2: Transforming growth factor beta receptor 2
TNFalfa: Tumor necrosis factor alpha
VEGF-A: Vascular endothelial growth factor A
